# Pegylation of charged polymer-photosensitiser conjugates: effects on photodynamic efficacy

**DOI:** 10.1038/sj.bjc.6601210

**Published:** 2003-08-26

**Authors:** M R Hamblin, J L Miller, I Rizvi, H G Loew, T Hasan

**Affiliations:** 1 Wellman Laboratories of Photomedicine, Massachusetts General Hospital, Boston, MA 02114, USA; 2 Department of Dermatology, Harvard Medical School, Boston, MA 02115, USA; 3 Department of Therapeutic Radiology, University of Vienna, Austria

**Keywords:** photodynamic therapy, photosensitiser, aggregation state, polyethylene glycol, polylysine conjugate, oxygen consumption

## Abstract

Conjugates between photosensitisers (PS) and charged polymeric carriers are under investigation for photodynamic therapy of cancer and may allow targeting to certain cell types or compartments in tumours. Covalent attachment of polyethylene glycol to macromolecules (pegylation) may alter their pharmacokinetics, cell type targeting, and photophysical properties. Macrophages may take up large amounts of aggregated PS, thus lessening the selectivity for cancer cells in tumours. We investigated the effect of pegylation on the uptake and phototoxicity of poly-L-lysine chlorin_*e6*_ conjugates with either cationic or anionic charges in two cell lines, human ovarian cancer cells and mouse macrophages. The cationic conjugate after pegylation became less aggregated, consumed less oxygen and had reduced cellular uptake. However, the phototoxicity corrected for cellular uptake increased three- to five-fold. In contrast, the anionic succinylated conjugate on pegylation became more aggregated, consumed similar amounts of oxygen, and had higher cellular uptake. The anionic conjugate showed the highest relative phototoxicity towards both the cell lines (compared to the other three conjugates) and it decreased most towards the macrophages after pegylation. Pegylation reduced the amount of oxygen consumed per chlorin_*e6*_ molecule when photosensitised cells were illuminated. These *in vitro* studies suggest that pegylation alters the phototoxicity of PS conjugates depending on the effect produced on the aggregation state.

Photodynamic therapy (PDT) is a novel approach for destruction of malignant or other unwanted tissue. It involves the administration of nontoxic dyes known as photosensitisers (PS) either systemically or topically, followed by illumination of the lesion with visible light (usually red) (Dougherty *et al*, 1998). The PS absorbs the light, and in the presence of oxygen transfers the energy, producing cytotoxic oxygen species (either singlet oxygen or oxygen radicals) (Ochsner, 1997). Our laboratory has studied the conjugation of PS to macromolecular delivery vehicles that are designed to improve their performance by increasing specificity and/or uptake in tumours or other pathological lesions, favourably altering pharmacokinetics or biodistribution (i.e. less skin photosensitivity) or decreasing phototoxicity to normal tissue (Hamblin *et al*, 1996,^1999^,^2000^; Duska *et al*, 1997; Del Governatore *et al*, 2000a,2000b; Molpus *et al*, 2000). The physical properties of macromolecular–PS conjugates such as size, ionic charge, hydrophobicity can be easily altered (Soukos *et al*, 1997), while keeping the same PS molecule joined to the conjugate that may or may not have a specific recognition site for a target (Hamblin *et al*, 1996). The conjugation of polyethylene glycol (PEG) to macromolecules has been advocated to increase serum half-life (Chinol *et al*, 1998; Delgado *et al*, 1992), reduce uptake by the reticuloendothelial system (Phillips *et al*, 1999), increase tumour accumulation (Tsutsumi *et al*, 1996), and increase water solubility (Greenwald *et al*, 1999). Polyethylene glycol has been attached to hydrophobic PS in order to increase their solubility (Hornung *et al*, 1997). Poly-L-lysine (pL) may be used as the macromolecular backbone for attaching PS such as chlorin_*e6*_ (c_*e6*_) to some of the *ɛ*-amino groups of the polyamino-acid backbone, and in addition the unaltered *ɛ*-amino groups remain available to first attach PEG, and then the overall charge can be made anionic by succinylation (Soukos *et al*, 1997). We have shown that cationic and succinylated pL–c_*e6*_ conjugates have differing cellular uptakes and relative phototoxicities *in vitro* (Soukos *et al*, 1997) and different biodistributions *in vivo* (Duska *et al*, 1997). It was shown that there were also differences in the rate at which these charged polymer–PS conjugates left the tumour vasculature as revealed by *in vivo* fluorescence microscopy (Hamblin *et al*, 1999). Polycationic molecules are known to be rapidly and efficiently taken up by cells through endocytosis (Basu *et al*, 1976), but have the drawback that they are also cleared rapidly from the systemic circulation *in vivo* (Vorbrodt *et al*, 1996), and pegylation might increase the serum half-life, thus improving the tumour localising properties of these molecules. We recently reported (Hamblin *et al*, 2001) on the effect of pegylation on the uptake and phototoxicity of a neutral acetylated pL–c_*e6*_ conjugate. It was found that pegylation reduced the tendency of the conjugate to aggregate, reduced its cellular uptake, and increased the phototoxicity towards cancer cells while the phototoxicity towards macrophages was decreased. Conjugates were injected i.p. into nude mice bearing i.p. OVCAR-5 tumours, and the pegylated conjugate gave higher amounts of PS in tumour and higher tumour : normal tissue ratios, and increased the depth to which the c_*e6*_ penetrated into the peritoneal wall. We now report on the effect pegylation of conjugates with either positive or negative charges has on the oxygen consumption and photodynamic efficacy against two cell lines, epithelial cancer cells and mouse macrophages.

## MATERIALS AND METHODS

### Cell lines

NIH:OVCAR-5 (OVCAR-5) cells were obtained from Dr T Hamilton (Fox Chase Cancer Institute, Philadelphia, PA, USA) and J774. A1 (J774) mouse macrophage-like cells were from ATCC (Rockville, MD, USA). Cells were grown in RPMI-1640 media containing HEPES, glutamine, 10% heat-inactivated fetal bovine serum, 100 U ml^−1^ penicillin, and 100-*μ*g ml^−1^ streptomycin, and maintained in an incubator at 37°C in a humidified atmosphere of 5% carbon dioxide.

### Preparation and analysis of conjugates

This was carried out essentially as previously described for pL-c_*e6*_-ac and pL-c_*e6*_-PEG-ac (Hamblin *et al*, 2001). Briefly, pL hydrobromide (degree of polymerization=46, mean MWt=5000, Sigma Chemical Co, St Louis, MO, USA) was reacted with c_*e6*_ *N*-hydroxysuccinimide ester (Soukos *et al*, 1997) in DMSO. After purification by exhaustive dialysis, the product was split into two equal parts. To one part was added methoxypolyoxyethylene imidazolyl carbonyl (average MWt=5000, Sigma) giving two solutions containing pL-c_*e6*_ and pL-c_*e6*_-PEG. Each of these was split into two equal parts, one of which was reacted with an excess of solid succinic anhydride and all the four preparations were then exhaustively dialysed as before. The optical densities at 400 nm measured in 0.1 M NaOH/1% SDS were similar as were the absorption spectra, and the extinction coefficients at 400 nm were assumed to be the same as c_*e6*_ (150 000 M^−1^ cm^−1^). Fluorescence calibration curves (excitation=400 nm, emission=668 nm) were constructed for each conjugate so that the fluorescence could be converted into mol c_*e6*_ equivalent. Pegylation was confirmed as previously described (Hamblin *et al*, 2001) by partition between two aqueous phases of PEG 8000 (4%) and dextran (MWt=500 000, 5%). The partition coefficients were calculated by dividing the fluorescence in the upper PEG phase by the fluorescence in the lower dextran phase. The aggregation of the conjugates as a function of concentration was measured as described previously (Hamblin *et al*, 2001). This was performed by preparing two series of serial two-fold dilutions of each conjugate (20 *μ*M–110 nM) in RPMI-1640 (containing 10% FCS). One set of dilutions was then centrifuged at 16 000 **g** for 15 min at 4°C, while the other set was gently agitated. The amount of conjugate in solution in each tube was determined by fluorescence and the fraction aggregated was calculated from the difference between the fluorescence in the supernatant of the centrifuged and that in the agitated samples.

### Oxygen consumption in solution

A LICOX oxygen partial pressure monitor (GMS, Kiel-Mielkendorf, Germany) was used to monitor the consumption of oxygen when the conjugates (2 *μ*M solutions in RPMI-1640 containing 10% FCS) were illuminated by 75-mW cm^−2^ 666 nm light in a 1-cm cuvette. After calibrating the Clarke electrode with pure N_2_ and pure O_2_, the conjugate solutions were oxygenated to approximately 500 mm*p*O_2_, the cuvette was then sealed and stirred by a Hellma Cuvet-O-Stir (Model 333, Forest Hill, NY, USA) and illumination commenced. The signal from the electrode was captured by LICOX software on a PC via a RS232 interface. The *p*O_2_ showed an exponential decline, and fluorescence measurements before and after illumination gave a measure of photobleaching. The experiment was repeated three times by adding fresh aliquots of each conjugate and reoxygenating. The initial rate of oxygen consumption was determined by drawing a tangent to the exponential *p*O_2_ decay curve.

### Cellular uptake

In total, 100 000 cells in 1-ml RPMI-1640 with 10% FCS were seeded into each well of 24-well culture plates. When the cells reached 80% confluency they received medium with 10% FCS containing 1 *μ*M c_*e6*_ equivalent and were incubated at 37°C for 4 h. Cells were washed and detached with trypsin–EDTA and the cell pellets were dissolved in 1.5 ml 0.1 M NaOH/1% SDS for at least 24 h to give a homogenous solution, and the fluorescence measured as previously described (Hamblin *et al*, 2000). The protein content of the entire cell extract was then determined by a modified Lowry method (Markwell *et al*, 1978) using bovine serum albumin dissolved in 0.1 M NaOH/1% SDS to construct calibration curves. The trypsin supernatant was also checked for the presence of fluorescence, which was negligible. Results were expressed as mol of c_*e6*_ per milligram of cell protein.

### Phototoxicity

In total, 25 000 cells in 0.1-ml RPMI-1640 with 10% FCS were seeded in each well of 96-well plates and cultured for 24 h until 70% confluent. Sextuplicate wells were given fresh complete medium containing the PS at a final concentration of 1 *μ*M c_*e6*_ equivalent for 4 h. Cells were washed twice, fresh medium was added and the cells exposed from beneath to 666 nm light delivered from an argon-ion pumped dye laser (Innova 100: CR-599 Coherent, Inc., Palo Alto, CA, USA) to give fluences ranging from 0 to 10 J cm^2^ at an irradiance of 50 mW cm^2^. Cells were then incubated with fresh medium for 24 h when the MTT-microculture tetrazolium assay was used to measure viability (Merlin *et al*, 1992). The survival fraction was calculated compared to dark controls incubated with conjugate for periods equal to irradiation times.

### Oxygen consumption in cells loaded with conjugate

In order to obtain sufficient c_*e6*_ in the cells to give a measurable consumption of oxygen, P100 plates with either J774 or OVCAR-5 cells were grown to near confluency and conjugates were added in the case of J774 at 2 *μ*M and OVCAR-5 at 4-*μ*M c_*e6*_ equivalent concentrations in complete medium for 24 h. The cells were then washed, trypsinised, and centrifuged to give a cell pellet, which was resuspended in 3-ml PBS and 2 ml of this was introduced into the stirred cuvette fitted with the LICOX electrode described above. Aliquots of the cell suspension were taken to determine the c_*e6*_ and cell protein concentrations as described. The cell suspension was illuminated with 75 mW cm^−2^ 666-nm light and the oxygen consumption trace recorded as described previously. After completion of illumination, aliquots of the cell suspension were taken to quantify photobleaching of the cellular fluorescence.

### Statistical methods

Differences between two means were evaluated by a two-sided unpaired Student's *t*-test assuming equal or unequal variances as appropriate. Standard deviations of the ratios of two means were obtained by calculating in quadrature (Taylor, 1982). *P*-values of less than 0.05 were considered significant.

## RESULTS

### Preparation and characterisation of pegylated conjugates

**Figure 1:**
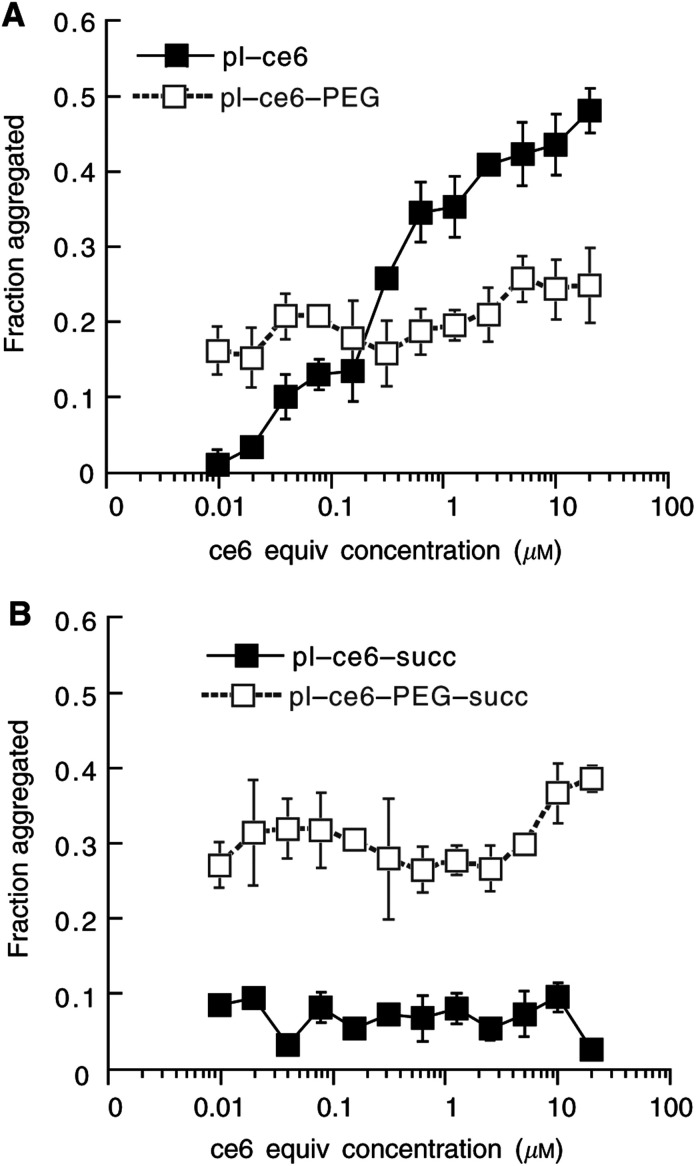
Effect of pegylation on the extent to which conjugates are aggregated at varying concentrations in serum-containing medium. (**A**) cationic pL–c_*e6*_ and pL–c_*e6*_–PEG, (**B**) anionic pL–c_*e6*_–succ, and pL–c_*e6*_–PEG–succ. Points are derived from the difference in fluorescence in 0.1 M NaOH/1% SDS between aliquots taken from samples centrifuged at 16 000 **g** at 4°C and from those agitated at room temperature. Each point is the mean of fluorescence determinations from three aliquots and error bars are s.d.

Since the substitution ratio (the number of c_*e6*_ molecules per polylysine chain) is likely to play a role in their photochemical and biological behaviour, the synthetic scheme used ensures that all four conjugates are directly comparable. The substitution ratio in this study was estimated by absorption spectroscopy to be 4 c_*e6*_ per chain of 46 lysine residues (8.7%). Two-phase partition in PEG 8000/dextran 500 mixtures showed increased partition coefficient after pegylation for both pairs of charged conjugates (pL-c_*e6*_: 1.95±0.07; pL-c_*e6*_-PEG: 3.93±0.02, *P*<0.0001; pL-c_*e6*_-succ: 1.24±0.07; pL-c_*e6*_-PEG-succ: 1.83±0.01, *P*<0.0001). There were no differences in absorption spectra between any of the conjugates whether measured in PBS or in NaOH/SDS (data not shown). In order to attempt to characterise differences in aggregation behaviour that could be visually observed between the various conjugates, experiments were performed in medium containing serum over three logs of dilution. The results are shown in Figures 1A and B

. The cationic conjugates aggregate in a concentration-dependent manner, the extent of which is reduced by attachment of PEG, especially at concentrations >100 nM. By contrast, the succinylated conjugate is hardly aggregated at any concentration, but when it is pegylated, the degree of aggregation is increased at all concentrations.

### Oxygen consumption in solution

Photochemical oxygen consumption by the various conjugates was measured by illuminating them at 2 *μ*M c_*e6*_ equivalent concentration in complete medium containing 10% FCS and a *p*O_2_ of approximately 500 mmHg. Each illumination lasted for approximately 12 min and was repeated three times for each conjugate. From the trace the initial rate of oxygen consumption could be determined in mmHg per minute by measuring the slope of a tangent drawn to the exponential curve, and the total amount of oxygen consumed (Hamblin *et al*, 2001). Fluorescence was measured before and after illumination in order to compare the extent of photobleaching between the conjugates. The means of the values of the three initial rates of oxygen consumption are shown in Table 1

**Table 1:** Oxygen consumption and photobleaching in solution

	**Initial rate O_2_ (mmHg min^−1^)**	**% Photobleaching**	**Mol O_2_ consumed per mol c_*e6*_**
pL–c_*e6*_	60.83±2.75	89±4	46±1
pL–c_*e6*_–PEG	44.47±2.77*	73±8*	39±3*
pL–c_*e6*_–succ	56.22±8.48	86±2	76±3
pL–c_*e6*_–PEG–succ	57.84±3.02	79±8	72±2

Conjugates were dissolved at a concentration of 2 *μ*M c*_e6_* equivalent in 2 ml 10% serum-containing medium at an initial *p*O_2_ of approx. 500 mmHg, in a stirred cuvette fitted with a LICOX oxygen probe. Illumination was carried out with 666 nm light at 75 mW cm^−2^. Initial rates were calculated from the slope of the tangent to the exponential curve of oxygen consumption and are expressed as mmHg per minute. Photobleaching was determined from the initial and final fluorescence in aliquots of the solution. Mol O_2_ consumed per mol c*_e6_* was determined from the total O_2_ consumption and the mol c*_e6_* consumed by photobleaching. Values are means (±s.d.) of triplicate determinations in two separate experiments.

* *P*<0.05 compared to non-PEG conjugate.

. The cationic pegylated conjugate had a significantly lower initial rate of oxygen consumption than the nonpegylated conjugate. In the case of the succinylated conjugate, however, pegylation made no significant difference to the rate of oxygen consumption. Similar results were seen in the percentage of-photobleaching after 12 min illumination with the non-PEG cationic being significantly more photobleached than the pegylated counterpart, but no significant difference between PEG and non-PEG succinylated conjugates. This implies that the reduction in oxygen consumption seen when the cationic conjugate is pegylated is not simply due to easier photobleaching. By dividing the number of moles of O_2_ consumed by photo-oxidative processes (assuming water at 25°C with *p*O_2_ of 740 mmHg is 1.27 mM O_2_; Weast, 1998) by the number of mol of c_*e6*_ consumed by photobleaching, the average number of molecules of O_2_ used by each c_*e6*_ molecule before being destroyed could be calculated. Pegylation of the cationic conjugate significantly reduced the number of O_2_ molecules consumed, while there was no significant difference for the succinylated conjugate.

### Cellular uptake

The cellular uptakes were compared between cell lines, between charges, and between PEG and non-PEG (Table 2

**Table 2:** Cellular uptake, oxygen consumption in cells and photobleaching of PEG and non-PEG conjugates.

	**Uptake (pmol c_*e6*_** **equivalent mg^−1^ cell protein)**		**Initial rate *p*O_2_ (mmHg min^−1^) per nmol c_*e6*_ in cell suspension**		**Mol O_2_ consumed per mol c_*e6*_**		**% Photobleaching**	
	**J774**	**OVCAR-5**	**J774**	**OVCAR-5**	**J774**	**OVCAR-5**	**J774**	**OVCAR-5**
pL–c_*e6*_	438±18*†§	215±27*§	20±8*†§	71±1§	385±9*†	124±48	71±1§	74±13
pL–c_*e6*_–PEG	112±22†§	80±3§	12±9†	73±12	146±90§	101±73	73±12	73±12
pL–c_*e6*_–succ	37±6*	28±5*	147±9*†	78±1	326±239*†	629±204*	78±1	53±20
pL–c_*e6-*_PEG–succ	64±18†	49±7	15±12†	67±17	61±36	77±61	67±17	66±15

Cells were incubated with the conjugates at 1 *μ*M c*_e6_* equivalent concentration (for uptake) or 2 *μ*M (J774) and 4 *μ*M (OVCAR-5) for oxygen consumption for 4 h at 37°C in 10% serum-containing medium. Cell pellets were dissolved in 0.1 M NaOH/1% SDS for fluorescence quantification of c*_e6_*, or suspended in PBS for oxygen measurements. An initial *p*O_2_ of approx. 300 mmHg was used in a stirred cuvette fitted with a LICOX oxygen probe. Illumination was carried out with 666 nm light at 75 mW cm^2^. Initial rates were calculated from the slope of the tangent to the exponential curve of oxygen consumption and are expressed as mmHg min^−1^ nmol^−1^ c*_e6_* in the 2 ml of cell suspension. Mol O_2_ consumed per mol c*_e6_* was determined from the total O_2_ consumption and the mol c*_e6_* consumed by photobleaching. Photobleaching was determined from the initial and final fluorescence in aliquots of the cell suspension. Values are means (±s.d.) of three independent experiments performed in triplicate.

* *P*<0.05 compared to PEG conjugate;

† *P*<0.05 compared to OVCAR-5 cells;

§ *P*<0.05 compared to conjugate with anionic charge.

). J774 cells take up more c_*e6*_ from every conjugate than OVCAR-5 cells (range 1.3–3.2 times). Both the cell lines took up many times more c_*e6*_ from the nonpegylated cationic compared to the non-PEG succinylated (7–12 times); however, this difference was much less pronounced in the pegylated conjugates (1.6–1.8 times). For both the cell lines, pegylation reduced the uptake from the cationic conjugate (in the case of OVCAR-5 to 37% and J774 to 25% of the non-PEG values). However, pegylation of the succinylated conjugates gave an opposite effect, increasing the cellular uptake in both the cell lines (in the case of OVCAR-5 to 175% and J774 to 173% of the non-PEG values).

### Phototoxicity

**Figure 2:**
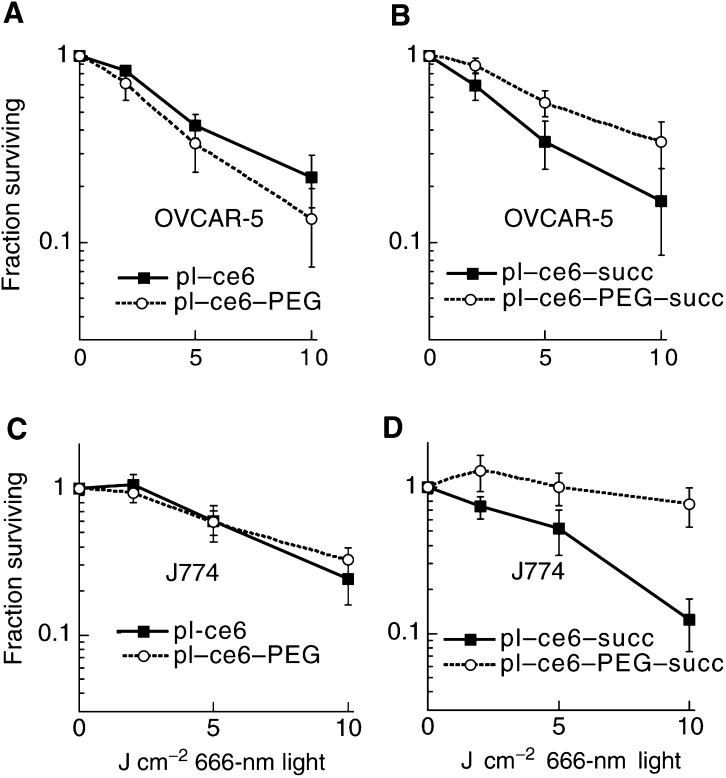
Phototoxicity curves comparing the light dose responses of the survival fractions of OVCAR-5 (**A**, **B**) and J774 (**C**, **D**,) cells incubated with (**A**, **C**) cationic pL–c_*e6*_ and pL–c_*e6*_–PEG, (**B**, **D**) anionic pL–c_*e6*_–succ and pL–c_*e6*_–PEG–succ. Cells were incubated for 3 h in serum-containing medium with conjugates added at 1 *μ*M c_*e6*_ equivalent concentrations. After illumination, cells were given fresh medium and 24 h later mitochondrial activity was determined by the MTT test. Survival fraction was calculated as the ratio of the 480 nm absorption from PDT-treated cells, to that from those given conjugate and kept at room temperature in the dark for the duration of the illumination. Points are the means from four separate experiments each containing six wells and bars are s.d.

The light dose-dependent loss of mitochondrial dehydrogenase activity after PDT of OVCAR-5 cells, which had been incubated with the four conjugates, is shown in Figures 2A and B

**Figure 3:**
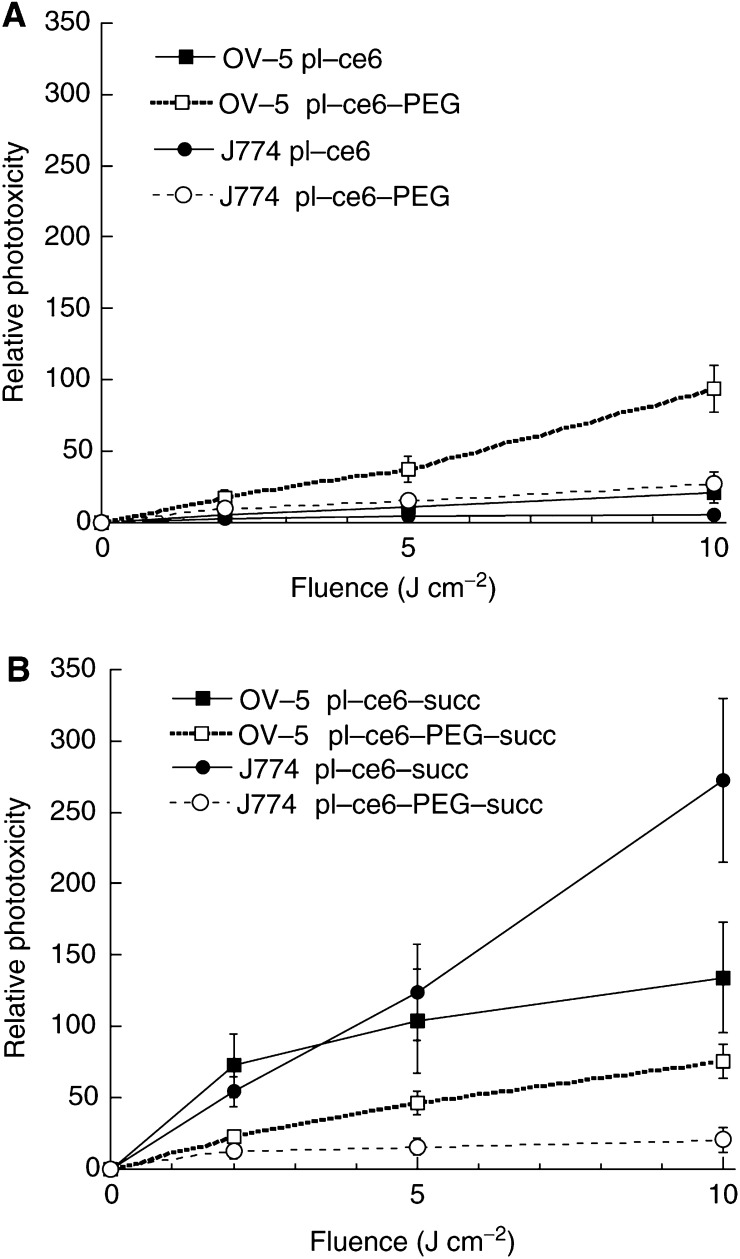
Relative phototoxicity curves comparing the light dose responses of the phototoxicities per nanomoles of c_*e6*_ taken up by the two cell lines (J774 and OVCAR-5) with (**A**) cationic pL–c_*e6*_ and pL–c_*e6*_–PEG, (**B**) succinylated pL–c_*e6*_–succ and pL–c_*e6*_–PEG–succ. Points were calculated from the reciprocal of the survival fraction (from Figures 2A to D) divided by the uptake in nanomoles c_*e6*_ equivalent per milligram cell protein (from Table 2). Error bars are the s.d. of the ratios calculated in quadrature.

. For the cationic conjugates, the pegylated conjugate is more phototoxic than the nonpegylated, while for the succinylated conjugate the reverse is true. Similar trends were observed for J774 cells where the cationic and cationic-PEG conjugates killed approximately equal amounts of cells, while the succinylated non-PEG conjugate killed significantly more cells than the pegylated counterpart (Figures 2C and D). In order to be able to compare the phototoxicities of the different conjugates corrected for differences in cellular uptake, the reciprocal of the survival fraction for each fluence was divided by the c_*e6*_ uptake in nanomoles c_*e6*_ per milligram cell protein. The resulting plots are shown in Figures 3A and B

. Despite the similar or higher absolute phototoxicity the cationic species showed compared to their anionic counterparts, when the killing data are corrected for uptake, it can be seen that the photodynamic efficacy per molecule is higher for the anionic species. For the cationic conjugates (Figure 3A), pegylation sharply increased the relative phototoxicity for both the cell lines (four-fold). Opposite results were found with the succinylated conjugates (Figure 3B); pegylation sharply decreased the relative phototoxicity towards both the cell lines, and most towards J774 cells (to less than one-tenth).

### Oxygen consumption in cells

The consumption of oxygen by a cell suspension loaded with conjugate was measured as the initial rate corrected for uptake of c_*e6*_ by the cells. The extent of photobleaching was determined by taking aliquots of cell suspension before and after illumination and extracting the fluorescence. The number of moles of oxygen consumed was divided by the number of moles of c_*e6*_ destroyed by photobleaching (calculated from the concentration of c_*e6*_ in the cuvette and the percentage of photobleaching). The results are shown in Table 2. Pegylation reduces the initial rates of O_2_ consumption per mole of c_*e6*_ taken up for all the conjugates in both the cell lines, and in addition reduces the number of molecules of oxygen consumed by each c_*e6*_ molecule for all the conjugates in both the cell lines. Note that the number of molecules of O_2_ consumed per c_*e6*_ when loaded into cells is significantly higher (up to 10 times) than the same number found when the conjugates were illuminated in solution for all the conjugates in both the cell lines except for pL-c_*e6*_-PEG-succ where it is similar (compare Table 1). Note also that the reduction in the rates of oxygen consumption caused by pegylation is more pronounced when the conjugates are loaded into cells than when they are in solution.

## DISCUSSION

This study has shown that the effect of pegylation on the photodynamic efficacy of PS conjugates depends both on the overall charge borne by the conjugate and on the target cell type. In the case of the cationic conjugate, pegylation can switch the selectivity away from macrophages towards cancer cells. It is known that the photophysical properties of tetrapyrrole PS are strongly affected by their aggregation state (Kessel, 1984), and it was expected that pegylation would affect the photodynamic process and this might be reflected in the oxygen consumption. In a recent study (Hamblin *et al*, 2001), we reported the effects of pegylation on an acetylated neutrally charged pL–c_*e6*_ conjugate, with regard to the aggregation-state, oxygen consumption, cellular uptake, localization, and phototoxicity.

In the present study, the cationic conjugate was less aggregated when pegylated than in the nonpegylated form (similar to the neutral conjugated studied previously; Hamblin *et al*, 2001), but surprisingly, the succinylated conjugate became more aggregated when pegylated. A possible explanation of this latter finding is that carboxylic acid groups of the polysuccinylated conjugate are ionised at pH 7.4 and electrostatic repulsion is responsible for the nonaggregated state of the non-PEG conjugate. After pegylation, the PEG chains with their associated water molecules may shield the charged carboxyl groups, thus lessening the mutual repulsion and encouraging the c_*e6*_ molecules to self-associate in a manner typical of tetrapyrroles.

The consumption of oxygen by photochemical oxidation mechanisms when PS are illuminated in the presence of an oxidizable substrate is well known (for a comprehensive review, see Ochsner, 1997). The Type II mechanism involves formation of excited state singlet oxygen by energy transfer from the triplet PS, and subsequent reaction with biomolecules. The PS returns to the ground singlet state and can repeat the process many times. The alternative Type I mechanism proceeds through initial electron transfer to or from the PS triplet state producing a radical cation or radical anion, which then further reacts with substrate and oxygen producing reactive oxygen species. In this case, the PS can react once only. It is generally thought that aggregated PS are less photoactive, that is, they are less fluorescent, produce less triplet state PS as determined by laser flash photolysis, and generate less singlet oxygen in solution (Redmond *et al*, 1985). It has been shown that the mechanism of haematoporphyrin photooxidation in liposomes changes from Type II to Type I on raising the concentration of the porphyrin which leads to aggregation (Grossweiner *et al*, 1982).

Pegylation of the cationic conjugate appears to reduce the degree of aggregation and also reduces the consumption of oxygen when illuminated in serum-containing medium. In the case of the succinylated conjugate where pegylation increases the aggregation, the oxygen consumption is not affected. However, the centrifugation experiments only measure aggregation on a scale of intermolecular particle formation, while the photophysics will almost certainly be affected by the intramolecular conformation of the tetrapyrrole molecules bound to the pL backbone, which could be altered by pegylation. Differences observed in oxygen consumption in solution could also be due to differences in the extent of binding of the conjugates to serum proteins.

Pegylation *reduced* both the degree of aggregation, and the cellular uptake of the cationic conjugate in both the cell lines (similar to the neutral conjugate studied previously (Hamblin *et al*, 2001)). However, pegylation *increased* the degree of aggregation and the uptake of the succinylated conjugate by both the cell lines. Two explanations are possible for the positive correlation between degree of aggregation and cellular uptake. Firstly, larger microaggregates may be endocytosed by macrophages; however, the fact that a similar effect was observed in OVCAR cells would make this hypothesis surprising. Secondly, the cationic conjugate may be endocytosed after binding to the plasma membrane through electrostatic interactions and triggering the formation of noncoated pits. This binding would be reduced by pegylation, due to its attached water layer acting as a buffer between the opposite charges. The repulsion that exists between the anionic regions on the plasma membrane and the carboxyl groups of the succinylated conjugate are responsible for the low uptake of this conjugate in its nonpegylated form, would similarly be reduced by pegylation and this would allow the molecules to approach much closer and increase the uptake by fluid-phase endocytosis. However, due to the absence of stimulation of endocytosis by the positive charges, neither anionic conjugate gave the uptake found with the cationic conjugates (both PEG and non-PEG).

Pegylation of the cationic conjugate led to increased killing of OVCAR cells, but slightly less killing of J774 cells compared to the non-PEG cationic conjugate. However, pegylation of the succinylated conjugate decreased the killing for OVCAR cells and decreased it even further for macrophages (Figures 2A–D). When the phototoxicity was corrected for differences in cellular uptake, it was found that pegylation of the cationic conjugate increased the relative phototoxicity most for OVCAR cells and led to a smaller increase for J774 cells, but pegylation of the anionic conjugate decreased the relative phototoxicity most for J774 cells and led to a smaller decrease for OVCAR cells. In contrast to previous studies (Hamblin *et al*, 2001) where pegylation altered the subcellular localisation of a neutral acetylated pL–c_*e6*_-ac conjugate (lysosomal before pegylation, extralysosomal after pegylation), in the present study no consistent differences were observed between pegylated and nonpegylated conjugates in either cell line (data not shown). Pegylation reduced both the initial rate and the total number of oxygen molecules consumed for both the charges in both the cell lines. The cationic conjugates consumed more oxygen in J774 cells than OVCAR cells, while the opposite was true for the anionic conjugates that consumed more oxygen in OVCAR cells than J774 cells. The drop in oxygen consumption was much steeper after pegylation for the anionic conjugates than for the cationic conjugates. However, the changes in phototoxic efficacy did not seem to correlate with either the rate or amount of oxygen consumption inside cells as shown in Table 2. The increase of selective phototoxicity towards cancer cells while sparing macrophages may have some relevance to clinical practice of PDT where preserving the function of tumour-associated macrophages may be desirable in facilitating the immune response element of the treatment (Korbelik *et al*, 1997). Tumour-associated macrophages have been reported to accumulate large amounts of PS *in vivo* and this is also likely to apply to macromolecular–PS conjugates (Korbelik and Krosl, 1996).

In conclusion, pegylation of a cationic pL–PS conjugate reduced its tendency to aggregate, decreased its cellular uptake, decreased the oxygen consumption both in solution and when loaded into cells, and increased the relative phototoxicity. These results were similar to those previously found with pegylation of the neutral acetylated conjugate in our previous study (Hamblin *et al*, 2001). Pegylation of the anionic succinylated conjugate had opposite effects, increasing the aggregation and cellular uptake but decreasing the relative phototoxicity. Experiments are underway in our laboratory to explore the effect of pegylation on the biodistribution, pharmacokinetics, and tumoricidal properties of these conjugates.
